# Sensitivity of latex agglutination faecal occult blood test in the Florence District population-based colorectal cancer screening programme

**DOI:** 10.1038/sj.bjc.6603759

**Published:** 2007-04-24

**Authors:** G Castiglione, C B Visioli, S Ciatto, G Grazzini, A G Bonanomi, T Rubeca, P Mantellini, M Zappa

**Affiliations:** 1 Diagnostic Imaging Unit, CSPO, Viale A, Volta 171, Florence 50131, Italy; 2 Epidemiology Unit, CSPO Via di San Salvi 12, Florence 50135, Italy; 3 Screening Unit, CSPO Viale A, Volta 171, Florence 50131, Italy; 4 Laboratory Unit, CSPO Via Cosimo il Vecchio 2, Florence 50139, Italy

**Keywords:** colorectal cancer, screening, faecal occult blood tests, sensitivity, proportional incidence method

## Abstract

We evaluated the sensitivity for colorectal cancer (CRC) of the latex agglutination test (LAT), an immunochemical test routinely used in the Florence District screening programme since 2000. Sensitivity was calculated by the proportional interval cancer incidence method in a population of 27 503 consecutive subjects screened in 2000–2002, interval cancers being identified by linkage to the Tuscany Cancer Registry files. Sensitivity was calculated overall and by gender, age, time since last negative LAT, CRC site, and rank of screening. Overall 1- and 2-year sensitivity estimates were 80.7 and 71.5%, respectively, suggesting that faecal occult blood testing screening sensitivity may be suboptimal due to testing or programme quality problems. Increasing screening sensitivity might be achieved if the detection rate of advanced adenomas could be increased without unacceptable loss in specificity.

Screening for colorectal cancer (CRC) by faecal occult blood testing (FOBT) has been effective in reducing CRC mortality and incidence (Towler *et al*, 2000), and is recommended in several countries as current health policy. Randomised trials demonstrating FOBT screening effectiveness used a 3-day guaiac-based test (G-FOBT), a chemical test based on haemoglobin peroxidase-like activity whose accuracy is affected by dietary factors such as the presence of nonhuman haemoglobin and peroxidases in vegetables, or by certain drugs, particularly NSAIDs. Sensitivity of G-FOBT for CRC has been reported to be as low as 43–66%, based on a 2-year screening interval (Jensen *et al*, 1992; Launoy *et al*, 1997; Moss *et al*, 1999; Jouve *et al*, 2001); attempts to increase sensitivity either by rehydration (Church *et al*, 1997) or by increased reagent concentration (Petrelli *et al*, 1994; Allison *et al*, 1996) were associated with unacceptable loss in specificity. Several studies have suggested that immunochemical FOBT (I-FOBT) is more sensitive and specific than G-FOBT (Castiglione *et al*, 1997; Saito *et al*, 2000; Zappa *et al*, 2001; Levi *et al*, 2006; Guittet *et al*, 2007), requiring no dietary restrictions, and might substantially improve screening cost effectiveness (Castiglione *et al*, 1997; Saito *et al*, 2000). Further progress in I-FOBT use was made by the introduction of the latex agglutination test (LAT), a quantitative and fully automated test (Yamamoto *et al*, 1990), which made it possible to choose the positivity cutoff values to optimise the balance between sensitivity and specificity, though the debate about the optimal cutoff point continues (Itoh *et al*, 1996; Castiglione *et al*, 2000; Nakama *et al*, 2001; Edwards, 2005; Vilkin *et al*, 2005). Sensitivity of G-FOBT and a previously used I-FOBT (reversed passive haemagglutination (RPHA)) for CRC, based on proportional interval cancer incidence, was determined previously (Zappa *et al*, 2001). In the present study we have evaluated LAT sensitivity using similar methodology.

## POPULATION AND METHODS

### Setting

A population-based FOBT screening programme has existed in Florence District since 1982, run by the Istituto Scientifico per la Prevenzione Oncologica (CPSO), its efficacy in reducing CRC mortality supported by a case–control study (Zappa *et al*, 1997). Reversed passive haemagglutination was used to replace G-FOBTin 1995 (Castiglione *et al*, 1997). A further study (Castiglione *et al*, 2000) showed that LAT (OC-Hemodia, developed with the OC-Sensor instrument, Eiken, Tokyo, Japan, and henceforth referred to as LAT) had a performance comparable with RPHA, and it was adopted as the standard test in our programme in January 2000, with a positivity threshold of 100 ng Hb ml^−1^ of sample solution. The main features and protocol have been reported in detail (Grazzini *et al*, 2004). Colonoscopy was recommended to FOBT-positive subjects.

### Population

Subjects aged 50–70, living in 19 municipalities in the Province of Florence, and attending FOBT screening from January 2000 to December 2002 were eligible for the present study.

Incident CRCs in 2000–2003 in Florence District residents were identified by linking the Tuscany Cancer Registry (http://www.cspo.it/REGISTRI//REGISTRO_RTT/rapporti/index.html) (by name, date, and place of birth), with screening archives. In case of partial matching (i.e. name and date of birth, not place of birth), manual assessment of records was performed. All CRCs diagnosed within 2 years after (1) a negative-LAT test or (2) a positive screen followed by a negative assessment, were regarded as interval cancers. Only interval cancers following a negative LAT were used to calculate ‘screening test sensitivity’, whereas both classes of interval cancers were used to assess ‘screening programme sensitivity’. In this way, the limits of the test were distinguished from those of the programme and the present figures compared with similar estimates in the literature.

In the sensitivity assessment, LAT-positive (LAT+) cases who refused assessment but had CRC diagnosed within 1 year since LAT were regarded as being detected by the programme, whereas cases with CRC diagnosed after 1 year were excluded. Screen detected and interval CRCs were classified according to bowel subsite and Dukes staging.

### Statistical analysis

Sensitivity was estimated by the proportional interval cancer incidence method (Day, 1985) which compares interval cancers within a given interval following a negative screening, or a positive test followed by a negative assessment, with the expected cancer incidence in the absence of screening (underlying incidence) according to the formula: 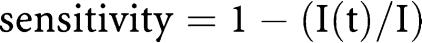 where I(t) is the observed interval cancers during interval *t* and I is the underlying incidence. Person-years at risk were calculated from the date of the first negative LAT. Observation ended (a) at 2 years, or (b) at interval cancer occurrence, or (c) at death from any cause, or (d) at the end of the study (December 2003), or (e) at the date of subsequent LAT if performed after an interval shorter than 2 years. Underlying CRC incidence was calculated using person-years and age-/sex-specific incidence rates provided by the Tuscany Cancer Registry (Table 1). Sensitivity was estimated both overall and by gender, age (50–59 or 60–70) at LAT, time since last negative LAT (first or second year of the interval), cancer location (rectum and rectosigmoid junction: ICDO=154; colon: ICDO=153), and rank of screening (first or subsequent). Confidence intervals (95% CI) were calculated on the basis of the exact Poisson's distribution, while statistical differences among strata (i.e. gender, age, site, time since last test, rank of screening) were tested by Gaussian approximation of the log likelihood (Clayton and Hills, 1993). Rate ratios (RRs) among strata were computed.

## RESULTS

From January 2000 to December 2002, 24 913 subjects performed 27 503 LATs, attendance rates in our programme being about 50% in the study period. Age/sex distribution of subjects at the time of the LAT are shown in Table 2. Table 3 presents LAT positivity and detection rates of colorectal lesions. Overall, LAT was positive in 1097 of 27 503 attending subjects (4.0%). Further diagnostic work-up was refused by 138 (12.6%) and accepted by 959 of 1097 LAT+ subjects. Colonoscopy was complete in 788 of the 959 (82.2%) and was incomplete in the remaining (17.3%). Double contrast barium enema was performed with incomplete colonoscopy in 106 of 166 (63.9%) subjects, and alone in five of 959 LAT+ cases (0.5%) not complying with colonoscopy and referred to their family physicians for diagnostic work up.

A total of 65 cancers were detected at screening, a detection rate of 2.4‰. Screen-detected CRCs’ Dukes stage was A, B, C, and D in 30, 16, 11, and six subjects respectively; two screen-detected neoplasms were carcinoids. Two cancers detected within 1 year from a positive-LAT case who had refused colonoscopy in our reference centres were regarded as screen-detected cancers. Advanced adenoma(s) (larger than 9 mm, or with high-grade dysplasia, or with villous component ⩾20%) was detected in 219 subjects.

For calculation of programme sensitivity 136 FOBT+ subjects refusing assessment were excluded; two of them had CRC detected after 1 year, and within 2 years of testing. Overall, 16 interval cancers were detected in the 2 years following a negative LAT. Dukes stage was A, B, C, and D in 3, 4, 3, and 4, respectively and was missing in two cases. Furthermore two interval cancers were detected within 2 years of a positive LAT followed by a negative assessment (1 Dukes B and 1 Dukes D).

Table 4 presents screening test sensitivity, person-years, expected numbers of cancers and sensitivity estimates overall and by time since last LAT, age, gender, location in the large bowel, or rank of screening. Overall, 2-year sensitivity was 73.8% (CI 57.4–85.0). Although no statistically significant difference was observed between sensitivity estimates (probably due to the small sample size), sensitivity was higher for subsequent ranks than at first screening (84.0 *vs* 57.3%, respectively, RR=2.27, 95% CI=0.97–7.34) and for elderly compared to younger subjects (79.5 *vs* 58.7%, respectively, RR=2.02, 95% CI=0.75–5.42). As expected, sensitivity in the first year was higher than that in the second year (82.9 *vs* 61.5%, respectively, RR=0.45; 95% CI=0.16–1.23). No difference was evident by gender or site.

Overall, 2-year programme sensitivity was 71.5% (CI 55.0–83.1), as shown in Table 5. No significant difference was evident by age, gender, site, or rank. Also, the difference in sensitivity between the first and second year (80.7 *vs* 59.2%, respectively, RR=0.47, 95% CI=0.18–1.22) was not significant.

## DISCUSSION

The present study was based on a relatively large series with cancer registry follow-up, allowing a reliable estimate of sensitivity according to the proportional interval cancer incidence method, probably the most reliable available. The traditional method (sensitivity=screen detected cancers/screen detected cancers+interval cancers) is open to criticism, as cancers with a long sojourn time and unlikely to surface clinically within 2 years, may be included among screen-detected cancers, particularly at prevalent screen, causing a lead time bias with overestimation of sensitivity. In fact, if we had used the traditional method the 1-year programme sensitivity would have been 91.6% (65/65+6) compared to our 82.9%, and the 2-year sensitivity 80.3% (65/65+16) compared to our 71.5%.

Nevertheless, our sensitivity method may be subject to selection bias. In fact, the method compares the incidence of interval CRC in compliers with the underlying incidence expected in the general population, assuming similar CRC incidence in compliers and noncompliers. Should this be greater in compliers, sensitivity in our study would be underestimated (Zappa *et al*, 1998). Unfortunately, we have been unable to get relevant information, and a study is in progress to verify the magnitude of any such bias by measuring the observed/expected CRC incidence ratio among noncompliers.

We regarded FOBT+ subjects who refused assessment but were diagnosed with CRC within 1 year of the positive screen as screen detected: in fact delayed assessment may still be motivated by (and ascribed to) the positive screen, but becomes unlikely after 1 year, whereas the probability of symptomatic CRC being diagnosed increases. The latter cases were therefore excluded when assessing programme sensitivity.

The sample size was limited, which may have prevented statistically significant results for age, gender, and bowel site analyses. Similarly, lack of statistical significance for sensitivity estimates based on time since last negative test or rank of screening may be due to the small number of events.

Although several studies have compared I-FOBT and G-FOBT (Petrelli *et al*, 1994; Allison *et al*, 1996; Saito *et al*, 2000), few have reported sensitivity estimates based on interval CRCs: Nakama *et al* (1996) reported values by the traditional method, of 90.9, 83.3, and 71.4% within 1, 2, and 3 years, respectively, using the 1-day Monohaem, immunological test. Zappa *et al* (2001) used the proportional interval cancer incidence method, and reported 1- and 2-year sensitivity estimates for CRC for 1-day RPHA testing of 89 and 82%, respectively, whereas corresponding estimates for 3-day G-FOBT were 64 and 50%, respectively. Using the traditional method, Launoy *et al* (2005) reported a 2-year sensitivity estimate of 85% for a RPHA-derived test (Magstream) with a low-positivity cutoff and a recall rate of 6%.

Latex agglutination test sensitivity in our study is higher than currently reported for unhydrated G-FOBT and slightly lower than in our previous study employing this method (Zappa *et al*, 2001) using RPHA I-FOBT as a screening test (80.0 *vs* 89% at 1-year and 71.5 *vs* 82.5% at 2-year interval, respectively) or in Launoy's study based on a similar automated analytical method. These results may reflect differences in analytical or statistical methods.

Other studies have assessed I-FOBT sensitivity using colonoscopy as the gold standard: Yoshinaga *et al* (1995) determined LAT sensitivity for CRC in 855 subjects undergoing colonoscopy; in 23 detected CRC, sensitivity estimates were 90 and 100% for Dukes A and B+ stages, respectively. Recently, Vilkin *et al* (2005) reported LAT colonoscopy-based sensitivity estimates of 66.7 and 100% with 1- and 3-day sampling, respectively. A large colonoscopy-based study of 1-day I-FOBT (Magstream) (Morikawa *et al* 2005) reported an overall sensitivity of 66%, estimates being stage – (advanced CRC>early CRC) and site dependent (left colon>right colon). Studies comparing FOBT results with colonoscopy for assessing FOBT sensitivity are not comparable with our study as they are open to lead time or overdiagnosis biases.

One possible factor negatively affecting screening programme sensitivity could be the suboptimal quality of the assessment phase. In our study two interval cancers were detected in FOBT+ subjects after a negative assessment, so even though we are not sure that CRC was present at the time of testing, these findings probably indicate weaknesses in our programme in the recall and assessment phases. Its sensitivity might be improved by referring FOBT+ subjects to selected centres with high endoscopy standards, to minimise the false-negative assessments, and by investing resources to improve compliance.

With respect to the screening test, annual rather than biennial testing would probably increase sensitivity by converting to ‘screen-detected’ most interval cancers occurring in the second year, but this would not necessarily represent higher efficacy on account of many more false–positives and unnecessary colonoscopies. In any case, this is hardly a realistic option in Florence or in other European countries given limited resources.

Lowering the positivity threshold (e.g. to 70 ngml^−1^) might have allowed the detection of two interval cancers with LAT between 70 and 100 ngml^−1^: if so, sensitivity would have been raised from 73.8 to 77.0% (or 75.4% with LAT between 80 and 99 ngml^−1^). Unfortunately, our study could not assess the corresponding increase in advanced adenoma detection. On the other hand, lowering the positivity cutoff to 70 or to 80 ngml^−1^ would have increased recall rate from 4 to 5.9% (1612/27 503) or to 5.1% (1397/27 503), respectively.

Multiple FOBT testing of 2–3 bowel movements has been proposed as another possible option to increase sensitivity, and 2-day testing has been suggested as the most cost-effective choice (Nakama *et al*, 1999; Yamamoto and Nakama, 2000). Lowering the positivity cutoff and doubling the number of sampled bowel movements, alone or combined, are presently being evaluated to increase screening accuracy in a multicentre study in Italy.

## Figures and Tables

**Table 1:** Incidence rates (per 100.000 person-years) of colorectal cancer in the Florence District by sex, age, and subsite in the period 1997–2002: Tuscany Cancer Registry

**Age (years)**	**Colon**	**Rectum**	**Overall**
*Males*			
50–59	59.6	40.2	99.8
60–69	146.3	75.7	222.0
70–74	241.6	115.1	356.7
			
*Females*			
50–59	42.9	21.0	63.9
60–69	87.6	39.6	127.2
70–74	135.6	56.4	192.0

**Table 2:** Age and sex distribution of subjects at the time they performed latex agglutination test

	**Males**		**Females**		**Total**	
**Age (years)**	***N***	**%**	***N***	**%**	***N***	**%**
50–59	6,580	50.0	7,198	50.1	13,778	50.1
60–69	6,134	46.7	6,677	46.5	12,811	46.6
70	431	3.3	483	3.4	914	3.3
						
Total	13,145	100.0	14,358	100.0	27,503	100.0

**Table 3:** Colorectal lesions detected of Latex Agglutination Test

	**No.**	**Rate**	**95% CI**
Screening tests	27503		
Positive tests	1097	4.0%	3.8–4.2
Screen positive subjects refusing assessment	138	12.6%	
Screen-detected cancers	65	2.36‰	1.8–3.0
No. screen-detected advanced adenomas	219	7.96‰	6.95–9.08
No. screen-detected cancer and advanced adenomas	284	10.3‰	9.16–11.59
			
*Interval cancers within 2 years*			
1 After a negative test	16	0.58‰	0.33–0.94
2 After a negative test or a positive test followed by negative assessment	18	0.65‰	0.39–1.03

**Table 4:** Latex test sensitivity

	**Person-years**	**Observed interval cancers**	**Expected cancers**	**2-year sensitivity (1-O/E) (%)**	**95% confidence intervals**
*Time since last test (months)*					
0–11	26,325	6	34.99	82.9	62.2–93.7
12–23	18,490	10	25.99	61.5	29.2–81.5
					
*Age*					
50–59	20,939	7	16.95	58.7	14.9–83.4
60–70	23,876	9	44.03	79.6	61.2–90.6
					
*Gender*					
Males	21,293	8	37.01	78.4	57.4–90.7
Females	23,522	8	23.96	66.6	34.2–85.6
					
*Subsite*					
Colon	44,815	11	40.30	72.7	51.2–86.4
Rectum	44,815	5	20.67	75.8	43.5–92.1
					
*Rank of screening*					
First	17,528	10	23.44	57.3	21.5–79.5
Subsequent	27,287	6	37.54	84.0	65.2–94.1
					
**Total**	**44,815**	**16**	**60.98**	**73.8**	**57.4–85.0**

Person-years, observed interval cancers, expected colorectal cancers and screening sensitivity by time since last test, age groups, gender, site, and rank of screening, according to a 2-year period of observation following a negative latex test.

**Table 5:** Programme sensitivity

	**Person-years**	**Observed interval cancers**	**Expected cancers**	**2-year sensitivity (1-O/E) (%)**	**95% confidence intervals**
*Time since last test (months)*					
0–11	27,220	7	36.29	80.7	60.3–92.2
12–23	19,127	11	26.96	59.2	27.0–79.6
					
*Age*					
50–59	21,552	8	17.45	54.2	9.7–80.2
60–70	24,795	10	45.78	78.2	59.8–89.5
					
*Gender*					
Males	22,108	8	38.52	79.2	59.1–91.0
Females	24,239	10	24.72	59.6	25.6–80.6
					
*Site*					
Colon	46,347	13	41.80	68.9	46.8–83.4
Rectum	46,347	5	21.44	76.7	45.6–92.4
					
*Rank of screening*					
First	18,232	10	24.48	59.2	24.9–80.4
Subsequent	28,115	8	38.76	79.4	59.3–91.1
					
**Total**	**46,347**	**18**	**63.24**	**71.5**	**55.0–83.1**

Person-years, observed interval cancers, expected colorectal cancers and screening sensitivity by time since last test, age groups, gender, subsite, and rank of screening, with a 2-year period of observation following a negative latex test or a positive-latex test followed by a negative assessment.
